# Running-Related Musculoskeletal Injuries in Saudi Arabia: Epidemiology, Biomechanical Determinants, and Global Comparative Insights

**DOI:** 10.3390/healthcare14172776

**Published:** 2026-09-01

**Authors:** Marwan M. A. Aljohani

**Affiliations:** Department of Physical Therapy, College of Medical Rehabilitation Sciences, Taibah University, Madinah 42353, Saudi Arabia; mmjohani@taibahu.edu.sa

**Keywords:** running-related injuries, musculoskeletal injuries, biomechanics, epidemiology, Saudi Arabia

## Abstract

**Background/Objectives:** Running is widely promoted for its health benefits, but it is also associated with a substantial burden of musculoskeletal injuries, particularly affecting the lower extremities. In Saudi Arabia, participation in recreational running has increased substantially in recent years. This review synthesizes global evidence on running-related musculoskeletal injuries and critically examines its applicability to the Saudi context, with emphasis on regional evidence gaps and research priorities. **Methods:** A narrative literature search of PubMed/MEDLINE and Scopus was conducted from database inception to August 2026 using predefined search concepts and eligibility criteria related to running, musculoskeletal injury, biomechanics, training load, and Saudi Arabia, with reporting guided by the SANRA criteria for narrative reviews. **Results:** Global evidence indicates that running-related injuries reflect complex interactions among training load, biomechanics, tissue capacity, and environmental factors. Injury prevalence and incidence vary considerably across studies because of methodological heterogeneity, although novice runners generally experience greater injury risk and the knee, lower leg, ankle, and foot are frequently affected. Saudi runner-specific evidence remains limited, with much of the available regional literature derived from cross-sectional, self-reported, or broader physically active populations. **Conclusions:** In Saudi Arabia, runner-specific injury data remain limited. Existing studies mainly concentrate on general physically active populations and lack standardized injury definitions, exposure-adjusted incidence measures, and prospective designs. Future research should prioritize prospective biomechanical surveillance using wearable sensor technology to capture real-world running mechanics and training loads.

## 1. Introduction

Running is one of the most common and accessible forms of physical activity worldwide [1]. It requires minimal equipment, can be performed in many environments, and provides well-established cardiovascular, metabolic, and mental-health benefits [1]. For these reasons, running is frequently promoted in public health initiatives aimed at reducing physical inactivity. However, regular participation in running is also associated with a high risk of musculoskeletal injury, which can limit long-term adherence and reduce the overall health benefits of exercise [2]. Running-related musculoskeletal injuries typically include overuse injuries, which develop gradually through repeated loading rather than acute trauma [3]. Current injury models emphasize that these injuries occur when the accumulated mechanical stress placed on tissues exceeds their ability to adapt and recover [4]. This process is influenced by multiple interacting factors, including training volume and intensity, movement mechanics, neuromuscular control, recovery, and individual biological characteristics [5]. As a result, no single risk factor can fully explain injury development. Despite extensive international research on running injuries, evidence from specific regional populations remains limited. Recent advances in wearable inertial sensors also enable field-based monitoring of running biomechanics and may facilitate the identification of movement alterations associated with injury risk.

In Saudi Arabia, participation in recreational running has increased in recent years, driven in part by national initiatives promoting physical activity [6,7]. Despite this growth, research specifically examining running-related injuries in Saudi populations remains limited. Most available studies focus on general physically active or gym-based samples and rely on cross-sectional designs. In addition, unique regional factors, such as extreme heat, urban running environments, and rapid transitions from sedentary lifestyles, are rarely considered [8,9,10,11]. This narrative review synthesizes global evidence on running-related musculoskeletal injuries and critically examines its relevance to the Saudi context, with the aim being to identify key knowledge gaps and inform future research and injury prevention efforts. Given the limited runner-specific evidence from Saudi Arabia, the contribution of this review lies primarily in contextualizing established global evidence, distinguishing direct from indirect regional evidence, and systematically identifying priorities for Saudi-specific research rather than establishing novel Saudi-specific injury determinants.

### Literature Search Approach

A narrative literature search was conducted in PubMed/MEDLINE and Scopus from database inception to August 2026. The core search strategy combined terms for the population/exposure (“running” OR “jogging”) with injury-related terms (“musculoskeletal injur*” OR “running-related injur*” OR “overuse injur*”) and topic-specific terms (“epidemiolog*” OR “biomechanic*” OR “training load” OR “risk factor*” OR “surveillance” OR “wearable*”). For Saudi-specific evidence, an additional search combined (“running” OR “runner*” OR “jogging” OR “physical activity” OR “exercise”) AND (“injur*” OR “musculoskeletal”) AND (“Saudi Arabia” OR “Saudi”), with syntax adapted as necessary to each database. Studies were included when they reported epidemiological, biomechanical, training-related, surveillance, or prevention data relevant to running-related musculoskeletal injuries, or provided Saudi-specific musculoskeletal injury evidence applicable to physically active populations where runner-specific evidence was unavailable. Original observational studies, prospective cohorts, systematic reviews, meta-analyses, and relevant scoping reviews were considered. Studies were excluded when they did not address musculoskeletal injury or running-related exposure, focused exclusively on non-musculoskeletal outcomes, or did not provide findings relevant to the scope of the review. Reference lists of relevant reviews and primary studies were additionally screened, and studies identified through this process were retained only when they met the same eligibility criteria. To reduce subjective literature selection, these predefined search concepts and eligibility criteria were applied consistently, with greater emphasis placed on systematic reviews, prospective studies, and runner-specific evidence where available; indirect Saudi evidence was explicitly identified as such in the synthesis. The review was prepared with consideration of the Scale for the Assessment of Narrative Review Articles (SANRA) criteria for narrative reviews [12].

## 2. Global Epidemiology of Running Injuries

### 2.1. Prevalence and Incidence of Running-Related Injuries Worldwide

Running-related musculoskeletal injuries are widely recognized as one of the most prevalent adverse outcomes associated with physical activity [13]. Epidemiological studies demonstrate substantial variability in reported injury rates, mainly attributable to heterogeneity in injury definitions, study designs, follow-up duration, and runner populations [14]. Systematic reviews and meta-analyses report global injury prevalence ranging from approximately 19% to 79%, highlighting the pervasive nature of running-related musculoskeletal injuries among recreational, competitive, and elite runners [15]. Incidence rates are commonly reported as injuries per 1000 h of running exposure, providing a standardized metric for cross-study comparison. A comprehensive meta-analysis by Videbæk et al. [16] estimated overall incidence rates between 2.5 and 12.1 injuries per 1000 h, with novice runners demonstrating the highest risk, followed by recreational runners, and comparatively lower rates among experienced or elite athletes [16]. This gradient may partly reflect differences in training adaptation, neuromuscular conditioning, and load-management experience between runner groups [17,18]. Longitudinal cohort studies further indicate that injury risk is highest during the initial months of running participation or following abrupt changes in training volume or intensity [18]. These findings are consistent with conceptual models in which overuse injury may develop when cumulative tissue loading exceeds adaptive capacity (Table 1).

### 2.2. Anatomical Distribution and Injury Patterns

Globally, running-related musculoskeletal injuries predominantly affect the lower extremities, accounting for more than 80% of reported injuries [19]. The knee is usually identified as the most frequently injured anatomical region, followed by the lower leg (including the tibia and Achilles tendon), ankle, and foot [20,21]. Common diagnoses include patellofemoral pain, medial tibial stress syndrome, Achilles tendinopathy, plantar fasciitis, and iliotibial band syndrome [22]. Studies show relatively similar anatomical injury patterns across continents, which is consistent with a contribution from running-related biomechanical loading, although this does not establish that biomechanics alone determines injury localization [25,31]. However, subtle variations have been observed based on running surface, footwear practices, and training environments, which may influence tissue-specific injury risk. Sex-based analyses indicate that female runners may exhibit a higher prevalence of hip and knee-related injuries, while male runners more frequently sustain calf and Achilles tendon injuries, although findings remain inconsistent across studies [26,27]. These differences are hypothesized to show sex-specific biomechanical, hormonal, and neuromuscular characteristics.

### 2.3. Influence of Runner Type and Training Characteristics

Epidemiological evidence generally indicates that injury rates differ by runner classification. Novice runners experience the highest injury incidence, with rates nearly double those of experienced runners [16]. Recreational runners represent the largest proportion of injured individuals globally due to their numerical dominance and variable training guidance, whereas elite runners, despite higher absolute training loads, often benefit from structured programs, professional supervision, and superior recovery strategies [32].

Training-related factors, including weekly mileage, sudden increases in training volume, running frequency, and inadequate recovery, are among the most reported predictors of injury in epidemiological studies [23,24]. Notably, no single safe training threshold has been universally identified, highlighting the importance of individualized load progression rather than absolute mileage limits.

### 2.4. Methodological Challenges in Global Injury Surveillance

Despite the extensive literature, significant methodological limitations complicate global comparisons of the epidemiology of running-related musculoskeletal injuries. Inconsistent injury definitions remain a central challenge, with some studies defining injury based on pain, some on time-loss, and others on medical consultation [28]. These discrepancies contribute substantially to the wide range of reported prevalence and incidence levels. Consequently, the consistency and strength of epidemiological associations should be interpreted cautiously, particularly when findings are derived from studies using different injury definitions, populations, and exposure measures. Additionally, many epidemiological studies rely on self-reported data, introducing recall bias and underreporting of minor or non-time-loss injuries. Heterogeneity may also arise from differences in biomechanical assessment protocols and the increasing use of wearable technologies for field-based measurement of movement and loading. Prospective surveillance studies with standardized definitions and exposure-adjusted outcomes are increasingly advocated as the gold standard for future research [29,30].

## 3. Running Injuries in Saudi Arabia

### 3.1. Physical Activity and the Emerging Running Culture in Saudi Arabia

Saudi Arabia has undergone a marked shift in physical activity participation over the past decade, largely driven by national public health initiatives under Saudi Vision 2030 [33]. These reforms have led to increased availability of organized sporting events, public running tracks, mass-participation races, and gym facilities. Running and jogging are now among the most commonly reported moderate-to-vigorous physical activities (MVPA), particularly among young and middle-aged adults [34,35]. Despite this growth, national surveillance data indicate that a substantial proportion of the Saudi population remains physically inactive, with many individuals transitioning rapidly from sedentary lifestyles to structured exercise programs [9]. This abrupt increase in activity exposure, particularly weight-bearing activities such as running, may elevate musculoskeletal injury risk, especially in novice runners with limited prior conditioning [8].

### 3.2. Participation Trends in Road-Running Events in Saudi Arabia

Participation in organized road-running events in Saudi Arabia has increased rapidly in recent years, highlighting national efforts to promote physical activity under Vision 2030 and the expansion of community sport initiatives led by the Saudi Sports for All Federation (SFA) and the Ministry of Sport. The Riyadh Marathon, the kingdom’s largest road race, illustrates this growth. The 2022 event attracted more than 10,000 participants from Saudi Arabia and internationally, marking a milestone in mass-participation sport in the country [36]. Participation rose to approximately 15,000 runners in 2023 and exceeded 20,000 in 2024, demonstrating sustained growth in community engagement. The 2025 event recorded over 40,000 participants from more than 130 countries, implying a national benchmark for mass-participation sport and highlighting the rapid expansion of recreational running in the kingdom. Beyond the Riyadh Marathon, community races and urban running initiatives in major cities such as Riyadh and Jeddah continue to attract thousands of participants, implying a widening base of recreational runners. This expansion has occurred in tandem with broader national reforms aimed at improving infrastructure efficiency, service delivery, and institutional coordination under Vision 2030, which may indirectly facilitate the organization and scaling of large public events, including community sporting activities [37]. Hence, these trends indicate a sharp rise in recreational running participation across Saudi Arabia. While this growth supports national public health goals, it also increases exposure to running-related musculoskeletal injuries, particularly among novice runners and individuals transitioning from sedentary lifestyles. Thus, the expanding popularity of mass running events highlights the need for injury surveillance systems and evidence-based prevention strategies tailored to the Saudi population.

### 3.3. Prevalence of Musculoskeletal Injuries in Physically Active Saudi Populations

Direct epidemiological studies specifically investigating running-related injuries in Saudi Arabia remain scarce. A recent runner-specific study conducted during a trail-running event in Riyadh evaluated 993 runners and recorded 109 medical encounters (11%). Lower-limb musculoskeletal injuries accounted for 15.6% of encounters, while muscle strain was the most common final musculoskeletal diagnosis, highlighting a measurable injury burden among Saudi endurance runners [38]. Importantly, this study represents a medical encounter cohort rather than an exposure-adjusted injury surveillance study; therefore, the reported 11% reflects the proportion of participants requiring medical attention and should not be interpreted as running-injury incidence per unit of exposure. However, several cross-sectional studies examining exercise-related and sports-related musculoskeletal injuries provide valuable indirect insights into the injury burden associated with running. A large cross-sectional study by Alrushud in 2022 of gym members in Saudi Arabia reported musculoskeletal injury prevalence ranging from 40% to 60%, with running and jogging reported among activities associated with lower-limb injuries [8]. The knee, ankle, and lower leg were the most frequently affected regions, mirroring global running injury patterns. Similarly, Alnasser et al. [9] in 2022 found that individuals engaging in endurance-type activities (including long-distance running) demonstrated higher rates of overuse injuries compared to those performing resistance-based exercise. More recent evidence from nationwide gym-based cohorts indicates that overuse injuries account for the majority of exercise-related musculoskeletal complaints, with knee pain, Achilles tendon disorders, and plantar heel pain being among the most prevalent conditions [39]. These studies describe injury patterns in general physically active populations, in which running represents one of several concurrent exposures and was not separately quantified. Their findings are consistent with a possible contribution from running-related loading but do not establish this relationship, as the injury burden attributable specifically to running cannot be isolated from these study designs.

### 3.4. Anatomical Distribution and Injury Characteristics

In Saudi-based studies, the anatomical distribution of musculoskeletal injuries appears broadly consistent with international running epidemiology. The knee emerges as the most commonly affected joint, followed by the ankle, foot, and lower back. Activities involving repetitive knee flexion and impact loading (such as jogging and running) have been reported as potential exposure factors [40,41]. Alwabli et al. examined exercise-related injuries among 247 female gym attendees in Al-Qassim Province. Nearly half of the participants reported sustaining an injury associated with physical activity. The ankle was the most frequently affected site (20.9%), followed by the knee (19.0%), while muscle injuries accounted for the majority of soft-tissue damage (55.7%). Although the study supports an association between exercise participation and injury occurrence, its findings are constrained by methodological limitations, particularly the restriction to a single gender and just one geographic region [42]. Given that these studies are largely cross-sectional, self-reported, and not restricted to runners, the predominance of lower-extremity injuries should be interpreted as broadly compatible with running-related loading rather than as evidence of a causal relationship. Similarly, the observed knee-related symptoms cannot be attributed specifically to training errors or biomechanical abnormalities without runner-specific prospective data.

### 3.5. Methodological Limitations of Saudi-Based Evidence

The current body of Saudi literature on musculoskeletal injuries is characterized by several notable limitations. Most studies are cross-sectional, rely on self-reported outcomes, and examine heterogeneous physically active populations rather than runners specifically [8,9]. Injury definitions are inconsistent, and exposure metrics such as weekly running distance, training intensity, or footwear characteristics are rarely reported. Critically, no prospective cohort studies have to date quantified running injury incidence per unit of exposure time (such as injuries per 1000 h of running) in Saudi Arabia. Similarly, there is a near-total absence of biomechanical investigations examining gait mechanics, loading patterns, or neuromuscular control in Saudi runners. Accordingly, associations derived from Saudi cross-sectional, self-reported, or non-runner-specific studies are interpreted throughout this review as indirect evidence and are not considered sufficient to establish runner-specific risk factors or causal relationships.

### 3.6. Environmental and Contextual Considerations

Unique regional factors may further influence running injury risk in Saudi Arabia. Extreme ambient temperatures, prolonged heat exposure, and limited seasonal variation may alter training behaviors, surface selection, and fatigue patterns. A further Saudi-specific consideration is the “vitamin D paradox”; despite abundant year-round sunlight, vitamin D deficiency remains prevalent in Saudi populations, which may have implications for bone health and susceptibility to bone-stress injuries, although runner-specific evidence remains limited [43,44]. Additionally, urban running environments characterized by hard surfaces may increase cumulative impact loading [8]. Given the extreme climate, treadmill running may represent an important indoor alternative in Saudi Arabia. Although treadmill and overground running are broadly biomechanically comparable, differences in some kinematic and kinetic variables should be considered when extrapolating injury-related findings between these settings [45]. Cultural factors, including limited access to structured coaching, delayed injury reporting, and reliance on self-guided training programs, may also be relevant, although their relationship with injury risk in Saudi runners has not been established. These contextual influences are rarely accounted for in existing studies, underscoring the need for culturally and environmentally customized research (Table 2). These Saudi-specific environmental and behavioral factors should therefore be regarded as plausible contextual modifiers rather than established injury determinants, because direct prospective evidence in Saudi runners remains limited.

## 4. Biomechanical Determinants of Running Injuries

Running-related musculoskeletal injuries are widely conceptualized as the result of repetitive mechanical loading exceeding the adaptive capacity of biological tissues. Biomechanical factors influence how external forces are generated, absorbed, and distributed in the musculoskeletal system during the running-gait cycle. These factors interact with training load, recovery, and individual tissue tolerance to determine injury risk [17]. From an epidemiological perspective, biomechanics represents one of the few modifiable intrinsic risk domains, making it a central target for injury-prevention strategies. However, identifying consistent biomechanical risk factors has proved challenging due to methodological heterogeneity and inter-individual variability.

Wearable inertial sensors provide an increasingly practical approach for assessing biomechanical alterations outside conventional laboratory settings. Such sensors have demonstrated sensitivity in detecting subtle postural-control deficits, including in the contralateral limb of individuals with chronic ankle instability, supporting their potential utility for identifying clinically relevant movement impairments [46]. Recent systematic-review evidence also indicates that associations between altered running biomechanics and running-related injuries remain variable, emphasizing the value of prospective biomechanical monitoring [25].

### 4.1. Kinematic Risk Factors

Kinematic variables describe joint and segment motion during running and have been extensively investigated as potential injury determinants. Reports indicate that excessive hip adduction, increased contralateral pelvic drop, and greater rearfoot eversion are among the most frequently reported kinematic features associated with running injuries, particularly patellofemoral pain and iliotibial band syndrome [17,47]. At the knee, increased frontal plane motion and altered sagittal plane mechanics have been linked to higher patellofemoral joint stress. Similarly, reduced ankle dorsiflexion during stance has been associated with Achilles tendinopathy and plantar fasciitis [48]. Nevertheless, effect sizes reported in studies are generally small to moderate, suggesting that isolated kinematic deviations alone are insufficient to cause injury.

### 4.2. Kinetic Factors and Loading Characteristics

Kinetic variables, including ground reaction forces (GRFs), loading rates, and joint moments, offer insights into internal tissue loading. Elevated vertical loading rates and impact peaks have been associated with bone-stress injuries and tibial stress fractures, particularly in novice and high-mileage runners [15,49]. Recent evidence emphasizes that how quickly force is applied (loading rate) may be more relevant than absolute force magnitude. Increased tibial acceleration and repetitive high-rate loading are thought to impair bone remodeling, increasing the susceptibility to stress-related injuries [49,50]. However, prospective evidence remains inconsistent, and several reviews caution against overinterpreting GRF metrics in isolation [25]. Thus, although several biomechanical parameters have been associated with injury, their predictive value remains uncertain and no single biomechanical marker can currently be considered a robust predictor of running-related injury.

### 4.3. Spatiotemporal Parameters and Gait Modifications

Spatiotemporal characteristics such as step rate (cadence), step length, and contact time have gained attention due to their modifiability. Increasing step rate by 5–10% has been shown to reduce knee-joint loading, hip adduction, and vertical GRFs, potentially lowering injury risk [51,52]. Systematic reviews of gait-retraining interventions indicate that targeted spatiotemporal modifications can produce favorable biomechanical changes and reduce pain in injured runners [53]. However, the long-term effectiveness of such interventions for primary injury prevention remains unclear, particularly in recreational populations.

### 4.4. Foot-Strike Pattern and Foot Mechanics

Foot-strike pattern (rearfoot, midfoot, forefoot) has been extensively debated as a biomechanical determinant of injury. Reports suggest that forefoot striking reduces impact loading rates but increases ankle plantar-flexor demand, potentially shifting injury risk from the knee to the ankle-Achilles complex [54,55]. Importantly, current evidence does not support a universal optimal foot-strike pattern for injury prevention. Instead, injury risk appears to depend on the interaction between strike pattern, training volume, footwear, and individual tissue capacity [25].

### 4.5. Fatigue, Neuromuscular Control, and Biomechanical Variability

Running-induced fatigue has been shown to alter lower-limb kinematics and plantar pressure distribution, often increasing joint-loading asymmetry and reducing shock-attenuation capacity [56,57]. These fatigue-related biomechanical changes may partially explain the higher injury incidence observed during periods of intensified training or competition [58,59]. Emerging evidence suggests that biomechanical variability, rather than rigidly ideal movement patterns, may be protective by distributing tissue load [60]. This perspective challenges traditional injury models and highlights the importance of adaptability in running mechanics. Overall, the evidence linking individual biomechanical variables to running-related injury remains inconsistent. Systematic reviews generally report small-to-moderate associations, with conflicting findings for cadence, foot-strike pattern, loading rate, and lower-limb kinematics. These variables should therefore be interpreted as potential contributors within a multifactorial injury process rather than as independent or reliable predictors of injury.

## 5. Risk Factors and Associated Determinants of Running-Related Injury

### 5.1. Multifactorial Nature of Running-Related Injury

Running-related musculoskeletal injuries arise from a complex interaction between biological, biomechanical, training-related, and environmental determinants. Contemporary injury models emphasize that no single risk factor is sufficient to cause injury in isolation. Rather, injuries occur when the cumulative load exceeds an individual’s tissue-specific tolerance. This multifactorial perspective is particularly relevant for recreational runners, whose training patterns, recovery strategies, and movement mechanics are often inconsistent. Risk factors are commonly categorized as non-modifiable (such as age, sex, injury history) and modifiable (including training load, biomechanics, footwear) [61]. Understanding their relative contribution is essential for targeted prevention strategies.

### 5.2. Non-Modifiable Risk Factors

One of the more consistently reported predictors of musculoskeletal issues in the running community is not footwear or surface, but rather an athlete’s own medical history. Multiple prospective cohort studies demonstrate that runners with a prior injury history have a 1.5–3-fold increased risk of sustaining a subsequent injury [18,62]. The mechanisms underlying this association remain uncertain but may include incomplete rehabilitation, residual biomechanical compensations, or premature return to high-intensity training before tissue remodeling is complete. For example, recreational runners who experienced an injury in the previous year are nearly twice as likely to be sidelined again, compared to those with a clean bill of health [63,64]. Consequently, injury-prevention strategies should prioritize specialized return-to-run protocols for previously injured athletes, rather than focusing solely on generic training-volume adjustments.

Evidence regarding age as an independent risk factor remains inconsistent. While some studies report higher injury rates among older runners due to reduced tissue elasticity and recovery capacity, others find no significant association after adjusting for training load [65]. On the other hand, sex-based differences in injury risk appear to be injury-specific rather than global. Female runners demonstrate higher incidence of hip- and knee-related injuries, whereas male runners more frequently sustain Achilles tendon and calf injuries [66,67]. These differences may reflect sex-specific anatomical, hormonal, and neuromuscular characteristics.

### 5.3. Training Load and Training Errors

Training-related factors are among the most frequently studied modifiable factors associated with running injury. Sudden increases in weekly mileage, intensity, or frequency, commonly referred to as training errors, have been associated with injury onset [24]. Prospective studies suggest that rapid progression in running distance is associated with higher injury risk, particularly among novice runners. However, absolute mileage alone appears to have limited predictive value, while the rate of change in load relative to prior conditioning may be more informative [18]. Inadequate recovery, insufficient rest days, and concurrent participation in multiple high-load activities have also been proposed as potential contributors to injury risk.

### 5.4. Biomechanical and Neuromuscular Factors

As discussed in Section 4, altered biomechanics influence internal tissue loading and injury susceptibility. Excessive hip adduction, increased knee valgus, elevated loading rates, and reduced shock attenuation have been associated with specific injury patterns [17,65]. Neuromuscular factors, including muscle weakness, impaired proprioception, and reduced movement variability, may limit the ability to adapt to repetitive loading, particularly under fatigue. Importantly, biomechanical risk factors often interact with training load; aberrant mechanics may only become injurious when combined with excessive or rapidly increased exposure.

### 5.5. Footwear, Surface, and Equipment-Related Factors

Footwear has received considerable attention as a potential injury determinant. While shoe type (neutral, cushioned, minimalist) may influence injury location, high-quality evidence indicates that footwear does not consistently alter overall injury incidence [66,68]. Barefoot running can alter foot-strike mechanics and redistribute lower-limb loading; however, evidence does not support a consistent injury-prevention benefit, and Saudi-specific evidence on barefoot running remains lacking [69]. In the Saudi context, outdoor barefoot running may also be limited by extreme heat and high surface temperatures, as sun-exposed asphalt and concrete in desert climates can reach temperatures capable of causing contact burns [70]. Improper shoe fit, excessive wear, and abrupt transitions between footwear types may, however, increase injury risk [71]. Running surface also plays a role, as harder surfaces are associated with increased impact loading, although the evidence remains mixed. Variability in terrain may be protective by distributing the mechanical stress in tissues [72,73].

## 6. Knowledge Gaps in Saudi Arabia

### 6.1. Limited Runner-Specific Epidemiological Data

Despite the rapid growth of recreational running in Saudi Arabia, there is a marked absence of runner-specific epidemiological studies. Although recent evidence from a Saudi trail-running event has provided runner-specific data, the broader Saudi literature still primarily focuses on general physical-activity populations, gym members, or team-sports athletes rather than dedicated longitudinal running cohorts [8,9,10,38,39]. Consequently, key epidemiological indicators, such as injury incidence per unit of exposure time, injury recurrence rates, and time-loss severity, remain undefined for Saudi runners. This lack of exposure-adjusted data prevents significant comparisons with global benchmarks and limits the ability to identify high-risk subgroups, such as novice runners or individuals transitioning from sedentary lifestyles.

### 6.2. Absence of Prospective and Longitudinal Research

Most Saudi-based studies employ cross-sectional designs, relying heavily on retrospective self-reporting of injuries. While valuable for estimating prevalence, such designs are insufficient for establishing temporal relationships between risk factors and injury development. To date, no prospective cohort studies have systematically tracked training load, biomechanical variables, and injury outcomes among runners in Saudi Arabia. International evidence demonstrates that prospective designs are essential for identifying causal risk factors and informing prevention strategies [18]. The lack of longitudinal Saudi data therefore represents a critical barrier to evidence-based injury prevention.

### 6.3. Scarcity of Biomechanical Investigations

Perhaps the most pronounced knowledge gap is the absence of biomechanical research on running in Saudi Arabia. While isolated studies have examined gait parameters in Middle Eastern populations or in non-running contexts, none have directly analyzed running kinematics, kinetics, or loading patterns in Saudi runners [74]. This gap is particularly concerning given the region’s unique contextual factors, including extreme heat exposure, the predominance of hard urban-running surfaces, and the high prevalence of obesity and delayed physical-activity adoption. Without biomechanical data, it is unclear whether injury mechanisms identified in Western populations are transferable to Saudi runners or whether region-specific adaptations exist. Wearable inertial sensors and field-based motion-analysis technologies could help address this gap by longitudinally tracking gait, loading, and movement patterns and linking potential pathomechanical alterations with injury surveillance.

### 6.4. Inadequate Consideration of Environmental and Climatic Factors

Environmental stressors, especially heat and dehydration, may influence running biomechanics and fatigue [75]. However, their relationship with running-related musculoskeletal injury risk has not been directly examined in Saudi runners. Moreover, to date, no studies have examined the interaction between climatic conditions and running-related musculoskeletal injuries in the region. International research suggests that heat stress can exacerbate neuromuscular fatigue, impair motor control, and subtly alter running kinematics, changes that could plausibly influence susceptibility to musculoskeletal injury, particularly during prolonged or high-intensity exercise [76]. In hot climates, elevated thermal strain may also compromise recovery and amplify the effects of training load on tissue stress [77]. The failure to contextualize environmental variables, such as ambient temperature and humidity, in Saudi injury research therefore represents a major omission that limits contextual interpretation and the development of climate-appropriate injury prevention strategies.

### 6.5. Lack of Standardized Injury Definitions and Surveillance Systems

Another key limitation is the absence of standardized injury definitions and national surveillance frameworks for sports and exercise-related injuries. Saudi studies frequently employ heterogeneous injury criteria, ranging from self-reported pain to medical consultation, complicating cross-study comparisons. Globally, consensus definitions for running-related injuries have been proposed [14], yet these have not been consistently adopted in Saudi research. Establishing standardized surveillance systems would greatly enhance data quality and comparability.

### 6.6. Underrepresentation of Female Runners

Although female participation in sports has increased substantially under Vision 2030, female runners remain underrepresented in Saudi musculoskeletal injury research. This gap limits our understanding of sex-specific injury mechanisms and risk profiles, which global evidence suggests may differ substantially [66,78]. Addressing this imbalance is essential for equitable and effective injury-prevention strategies.

## 7. Synthesis and Future Directions

**Figure 1:**
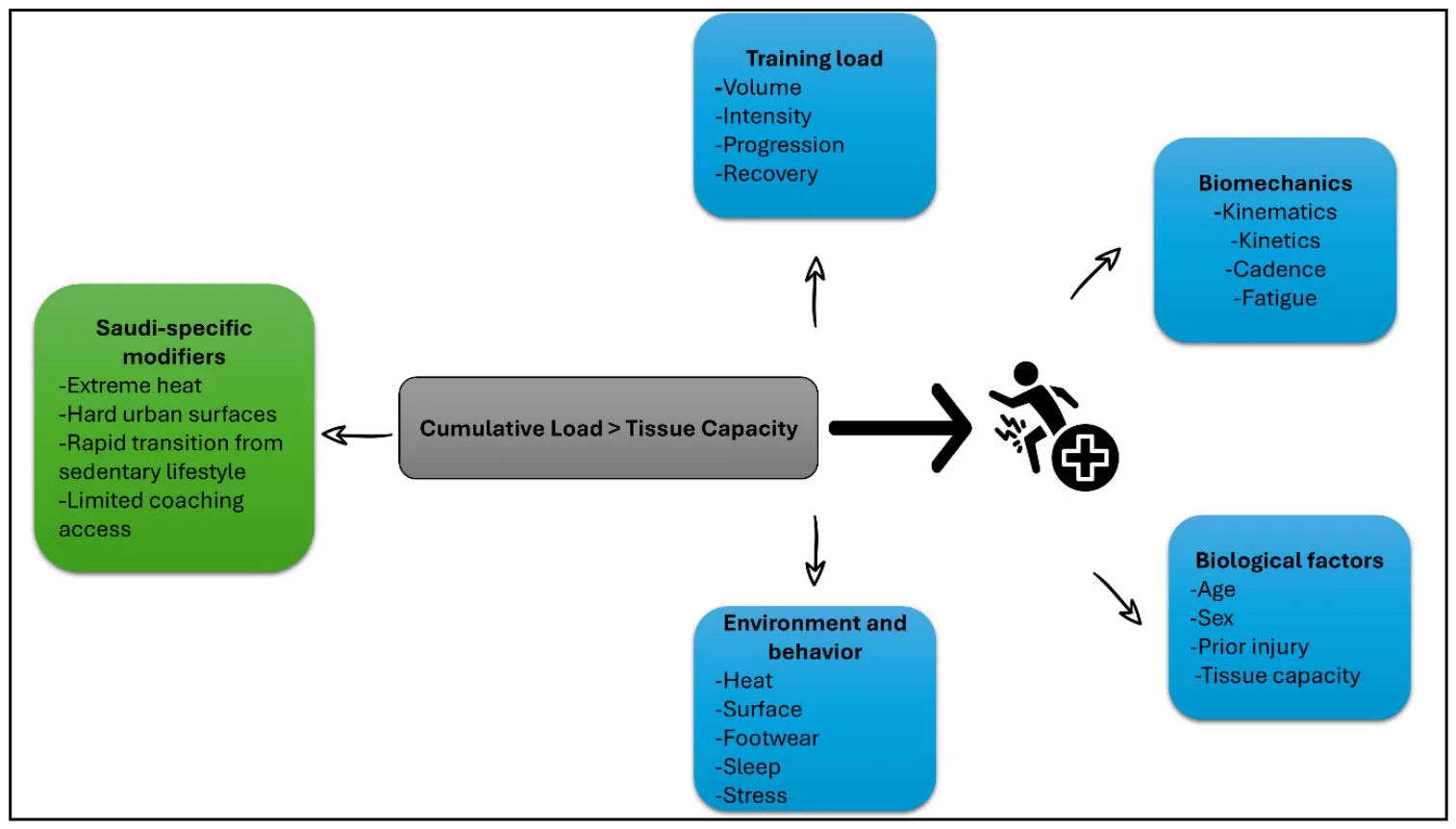
Contextual adaptation of established multifactorial models of running-related musculoskeletal injury development, incorporating Saudi Arabia-specific environmental and behavioral modifiers. This figure illustrates the multifactorial pathways through which running-related musculoskeletal injuries arise when cumulative mechanical load exceeds individual tissue adaptive capacity. Injury risk is shaped by the interaction of training load, biomechanics, biological characteristics, and environmental and behavioral factors. Saudi Arabia-specific modifiers, including extreme heat exposure, hard urban running surfaces, rapid transitions from sedentary lifestyles to structured running, and limited access to structured coaching, are highlighted to emphasize regional factors that may amplify tissue loading, fatigue, and injury susceptibility. The framework highlights the need for context-specific epidemiological surveillance, biomechanical investigation, and injury prevention strategies tailored to the Saudi running population.

Running-related musculoskeletal injuries remain a common problem globally, affecting runners at all levels of participation. International evidence shows high injury prevalence and incidence, predominantly involving the lower extremities, particularly the knee, lower leg, ankle, and foot. These injuries arise from a multifactorial interaction between training load, biomechanical characteristics, tissue capacity, and environmental exposure, rather than any single isolated risk factor. Injury risk increases when cumulative mechanical loading exceeds the ability of the subjects to adapt, particularly during periods of rapid training progression or inadequate recovery (Figure 1).

In Saudi Arabia, the rapid growth of recreational running under Vision 2030 has not been matched by an equivalent expansion of runner-specific research. Existing studies suggest a substantial burden of musculoskeletal injuries among physically active Saudi populations, with anatomical patterns broadly resembling those reported in runners internationally. However, because much of the Saudi evidence is cross-sectional, self-reported, and not runner-specific, these findings cannot establish the magnitude or determinants of running-related injury specifically. However, the absence of runner-specific epidemiological data, exposure-adjusted incidence metrics, standardized injury definitions, and prospective study designs limits sophisticated comparisons with international benchmarks and hinders the development of evidence-based prevention strategies. These gaps are particularly concerning given the region’s unique contextual factors, including extreme heat exposure, hard urban-running surfaces, and rapid transitions from sedentary lifestyles. Accordingly, the Saudi-specific conclusions of this review should be interpreted primarily as a contextual synthesis and identification of evidence gaps, rather than as evidence of distinct injury mechanisms or risk profiles in Saudi runners.

Future research should focus on prospective runner cohorts with standardized injury definitions and exposure-adjusted outcomes, alongside longitudinal monitoring of training load, recovery, and injuries (particularly among novice runners) in order to identify causal mechanisms. Such prospective studies should combine epidemiological surveillance with wearable biomechanical assessment in real-world settings to capture running mechanics and cumulative load, particularly under hot environmental conditions. Studies should also account for environmental factors, such as heat stress and dehydration, which may influence fatigue and biomechanics. In addition, randomized trials evaluating multifactorial injury-prevention strategies, including load management, neuromuscular strengthening, and gait modification, are needed in the Saudi context, along with ensuring adequate representation of female runners to support comprehensive and equitable injury-prevention frameworks.

## 8. Conclusions

Running-related musculoskeletal injuries are common and predominantly overuse in nature, with injury development reflecting interactions among cumulative mechanical loading, tissue capacity, training characteristics, biomechanics, and environmental factors. Global evidence indicates that the knee, lower leg, ankle, and foot are among the most frequently affected regions, with novice runners generally experiencing greater injury risk. In Saudi Arabia, however, runner-specific evidence remains limited, and much of the available regional literature is cross-sectional, self-reported, or derived from broader physically active populations. Consequently, the current Saudi evidence is insufficient to establish distinct injury determinants or risk profiles and should primarily be interpreted as contextual and hypothesis-generating. Prospective, runner-specific surveillance using standardized injury definitions, exposure-adjusted outcomes, and biomechanical assessment is needed to determine whether globally identified injury patterns and risk factors are directly applicable to Saudi runners and to support context-specific injury-prevention strategies.

## Figures and Tables

**Table 1:** Overview of running-related musculoskeletal injuries from global evidence.

Domain	Reference	Predominant Evidence Base	Key Findings
Injury prevalence	[15]	Systematic reviews/meta-analyses	Reported prevalence ranges widely from 20% to 80% among studies, mainly due to differences in injury definitions, study design, follow-up duration, and runner populations.
Injury incidence	[16]	Systematic reviews/meta-analyses of cohort studies	Incidence rates commonly range from 2.5 to 12.1 injuries per 1000 h of running exposure. Novice runners generally show the highest incidence, followed by recreational runners, with lower rates in experienced or elite runners.
Injury type	[3,4]	Systematic reviews/conceptual evidence	The majority of running-related injuries are overuse in nature, developing gradually due to repetitive mechanical loading, rather than acute trauma.
Anatomical distribution	[19,20,21]	Systematic reviews	>80% of injuries involve the lower extremities. The knee is most frequently affected, followed by the lower leg (tibia/Achilles), ankle, and foot.
Common diagnoses	[20,21,22]	Systematic reviews/prospective studies	Patellofemoral pain, medial tibial stress syndrome, Achilles tendinopathy, plantar fasciitis, and iliotibial band syndrome are the most frequently reported conditions.
Runner’s experience level	[16,18]	Systematic reviews/cohort studies	Injury risk is highest in novice runners, particularly during the first months of participation or following abrupt changes in training load. Recreational runners account for the largest absolute injury burden due to high participation numbers.
Key training-related risk factors	[18,23,24]	Systematic reviews/prospective cohort studies	Sudden increases in weekly mileage, intensity, or frequency; inadequate recovery; and high relative training load progression. No universal safe mileage threshold exists.
Prior injury	[18]	Systematic reviews/prospective cohorts	Previous injury is one of the most consistently reported non-modifiable predictors, with prospective studies reporting an approximately 1.5–3-fold increased risk of subsequent injury.
Biomechanical determinants	[15,17,18,25]	Systematic reviews/meta-analyses of prospective studies	Associations reported with excessive hip adduction, increased knee valgus, elevated loading rates, altered foot strike mechanics, fatigue-related kinematic changes, and reduced movement variability. Effect sizes are generally small to moderate.
Sex-related patterns	[20,21,26,27]	Systematic reviews/observational studies	Female runners show higher prevalence of hip and knee injuries, whereas male runners more frequently sustain Achilles tendon and calf injuries; findings are injury-specific and not universal.
Methodological limitations	[14,28,29,30]	Systematic/scoping reviews and surveillance-methodology studies	Heterogeneous injury definitions (pain-based, time-loss, medical attention), reliance on self-reporting, and limited prospective surveillance complicate cross-study comparisons.

**Table 2:** Comparison of running-related injuries based on evidence from global studies compared with Saudi Arabia.

Domain	Global Evidence	Saudi Arabia Evidence	Key Gap/Implication for Saudi Context
Evidence base (study focus)	Many studies specifically target runners (novice, recreational, elite), including cohort and surveillance studies.	Runner-specific studies are scarce; most studies assess general physically active populations (gym members), rather than dedicated runners.	Need runner-defined cohorts to avoid exposure misclassification and allow meaningful comparisons.
Study design	Increasing use of prospective cohorts with exposure tracking; systematic reviews/meta-analyses summarize the evidence.	Predominantly cross-sectional, retrospective self-reporting designs; prospective running cohorts are not reported.	Limits causal inference and prevents identification of temporal links between load/biomechanics and injury.
Injury definition	Heterogeneous (pain-based, time-loss, medical attention), but consensus efforts exist and are increasingly advocated.	Inconsistent definitions across studies; often self-reported pain/injury without standardized criteria.	Standardized definitions needed for comparability and surveillance quality.
Prevalence	Wide range (20–80%) depending on methods and runner type.	Indirect evidence suggests high musculoskeletal injury prevalence (40–60%) in active/gym populations; not consistently runners-only.	True running-specific prevalence is unknown; current estimates likely mix running with other activities.
Incidence (exposure-adjusted)	Commonly reported as injuries per 1000 h; meta-analytic estimates 2.5–12.1/1000 hrs, highest in novice runners.	No identified Saudi studies reporting injuries per unit of exposure time (per 1000 running hours).	Core epidemiologic metric missing; prevents benchmarking and monitoring over time.
Anatomical distribution	>80% lower extremities; knee most common, then lower leg/Achilles, ankle, foot.	Similar pattern reported in Saudi activity-based studies: knee and ankle commonly affected; lower extremities predominate.	Patterns appear comparable, but runner-specific localization and diagnosis-level detail remain limited.
Common diagnoses	PFP, MTSS, Achilles tendinopathy, plantar fasciitis, ITBS frequently reported.	Saudi studies often report body regions and broad injury categories; diagnosis-specific reporting is limited.	Need clinically anchored case definitions and diagnostic granularity to guide prevention/rehabilitation.
Runner type and risk profile	Novice runners at highest risk; early months and abrupt load changes are key risk windows.	Rapid transition from inactivity to structured exercise is plausible; however, runner-type stratification (novice vs. experienced) is rarely captured.	Identify high-risk subgroups (novice, returning-from-injury, obese/low-fitness) via runner cohorts.
Training load metrics	Load (volume, intensity, frequency) and rapid progression are frequently associated with injury; relative changes in load may be more informative than absolute mileage.	Training exposure variables (weekly distance, intensity, recovery, footwear transition) are rarely reported.	Incorporate digital logs/wearables to quantify load and detect risky progression patterns.
Biomechanical evidence	Extensive literature: kinematics (hip adduction/pelvic drop), kinetics (loading rates), spatiotemporal factors (cadence), fatigue-related changes; effect sizes small–moderate.	Few biomechanical studies assessing running-gait mechanics or loading in Saudi runners.	Priority gap: establish lab + field biomechanics (wearables) linked to injury surveillance.
Environmental context	Environmental factors increasingly considered; heat/fatigue may alter kinematics and risk; surfaces and footwear may shift injury location.	Extreme heat, hard urban surfaces, and limited seasonal variation are frequently mentioned but rarely measured or modeled.	Develop climate-aware surveillance (temperature/humidity), and context-specific prevention guidance.
Female representation	Many studies include both sexes; sex-specific patterns reported but not universal.	Female runners remain underrepresented; some Saudi studies are single-sex and region-limited.	Ensure recruitment of women and sex-stratified analyses to support equitable prevention strategies.
Surveillance systems	Standardized definitions and prospective surveillance increasingly advocated as best practice.	No national or standardized running-injury surveillance framework is described.	Implement surveillance aligned with consensus definitions and exposure-adjusted reporting.

ITBS, iliotibial band syndrome; MTSS, medial tibial stress syndrome; PFP, patellofemoral pain.

## Data Availability

No new data were created or analyzed in this study. Data sharing is not applicable to this article.

## Abbreviations

The following abbreviations are used in this manuscript:

GRFs	Ground reaction forces
MVPA	Moderate-to-vigorous physical activity
SFA	Saudi Sports for All Federation
